# The Hand That Protects: Structural Insights into a Porphyrin-Binding Protein

**DOI:** 10.1371/journal.pbio.0030167

**Published:** 2005-04-26

Eukaryotic cells have an organizational problem. The specialized proteins found in cellular organelles (structures with specific functions such as energy production) are mostly encoded within the nucleus. To build and maintain a cell that works efficiently under all conditions, each type of organelle needs to be able to send signals to the nucleus to say “Send more protein X” or “hold back on enzyme Y.” Think of it as the cellular version of grocery store clerks' restocking orders to the warehouse.

In plant cells, the chloroplasts (the photosynthetic organelles that convert light excitation energy into chemical energy) send signals to the nucleus to control the expression of the genes that encode chloroplast-localized proteins such as the enzymes that fix carbon dioxide, make chlorophyll, or perform photosynthesis. The accumulation of the chlorophyll precursor Mg-protoporphyrin IX provides one of these signals. A protein called GUN4 both enhances the activity of Mg-chelatase, the enzyme that makes Mg-protoporphyrin IX, and plays a role in the chloroplast-to-nucleus signaling activity of Mg-protoporphyrin IX in Arabidopsis, a well-studied plant.

To discover how GUN4 has these effects, Mark Verdecia in Joseph Noel's laboratory and Rob Larkin, formally in Joanne Chory's laboratory, determined the crystal structure of the GUN4 equivalent in the cyanobacteria Synechocystis. Cyanobacteria are the evolutionary ancestors of chloroplasts, so whatever GUN4 does in these cells is likely to be important in plant cells. The researchers' crystallographic studies, together with nuclear magnetic resonance and other studies, indicate that the porphyrin-binding region of Synechocystis GUN4 has a unique three-dimensional shape that resembles a cupped hand, the inner concave surface of which is highly hydrophobic. Because of this tendency to repel water, the researchers call this region the “greasy palm” of the cupped hand.

This structure suggests how GUN4 is involved in the chloroplast-to-nucleus signaling activity of Mg-protoporphyrin. By wrapping Mg-protoporphyrin IX in its cupped, greasy palm, the GUN4 structure provides a novel vehicle for binding Mg-protoporphyrin IX and may be involved in transporting signals from the chloroplast to the nucleus. In addition, the structure also suggests that GUN4 may be involved in photoprotection. Although light drives photosynthesis, which is essential to green plants, light has a downside—porphyrins combine with the oxygen released during photosynthesis to generate reactive oxygen species, which are generally damaging to the cell. GUN4, by cocooning Mg-protoporphyrin IX in its protective hand, may provide a way to safely move porphyrin around the chloroplast without exposing it to oxygen. Finally, the detailed structural and functional studies described by Noel and colleagues explain how GUN4 enhances the activity of Mg-chelatase. GUN4 binds Mg-protoporphyrin IX, the product of the chelatase, much better than protoporphyrin IX and so will tend to enhance the enzymatic reaction by removing its product.[Fig pbio-0030167-g001]

**Figure pbio-0030167-g001:**
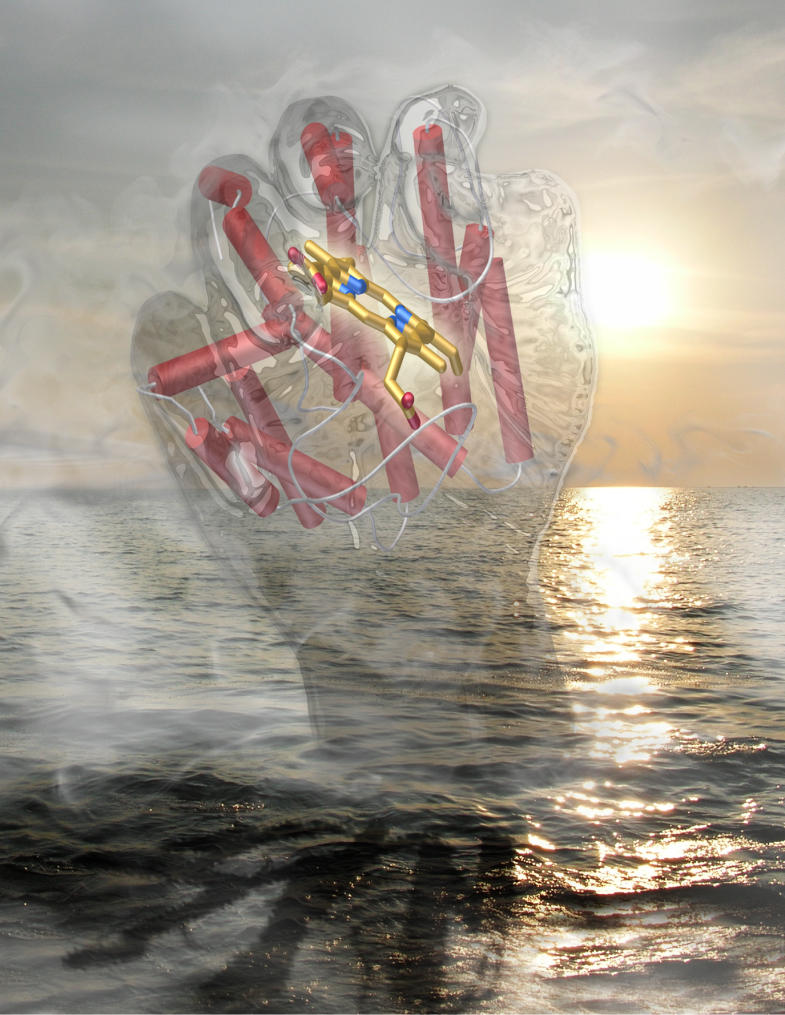
A “greasy palm” in the cupped hand of a porphyrin-binding protein reveals how this hydrophobic protein repels water. (Illustration: Mark Verdecia)

